# Fluorescence lifetime imaging of AMPA receptor endocytosis in living neurons: effects of Aβ and PP1

**DOI:** 10.3389/fnmol.2024.1409401

**Published:** 2024-06-10

**Authors:** Katie Prinkey, Emily Thompson, Junmi Saikia, Tania Cid, Kim Dore

**Affiliations:** Center for Neural Circuits and Behavior, Department of Neuroscience, School of Medicine, University of California at San Diego, La Jolla, CA, United States

**Keywords:** amyloid-beta, Alzheimer's disease, FLIM, live fluorescence imaging, superecliptic pHluorin, APP/PS1 neurons, primary hippocampal cultures

## Abstract

The relative amount of AMPA receptors expressed at the surface of neurons can be measured using superecliptic pHluorin (SEP) labeling at their N-terminus. However, the high signal variability resulting from protein overexpression in neurons and the low signal observed in intracellular vesicles make quantitative characterization of receptor trafficking difficult. Here, we establish a real-time live-cell assay of AMPAR trafficking based on fluorescence lifetime imaging (FLIM), which allows for simultaneous visualization of both surface and intracellular receptors. Using this assay, we found that elevating amyloid-beta (Aβ) levels leads to a strong increase in intracellular GluA1 and GluA2-containing receptors, indicating that Aβ triggers the endocytosis of these AMPARs. In APP/PS1 Alzheimer's disease model mouse neurons, FLIM revealed strikingly different AMPAR trafficking properties for GluA1- and GluA3-containing receptors, suggesting that chronic Aβ exposure triggered the loss of both surface and intracellular GluA3-containing receptors. Interestingly, overexpression of protein phosphatase 1 (PP1) also resulted in GluA1 endocytosis as well as depressed synaptic transmission, confirming the important role of phosphorylation in regulating AMPAR trafficking. This new approach allows for the quantitative measurement of extracellular pH, small changes in receptor trafficking, as well as simultaneous measurement of surface and internalized AMPARs in living neurons, and could therefore be applied to several different studies in the future.

## Introduction

Trafficking of α-amino-3-hydroxy-5-methyl-4-isoxazolepropionic acid (AMPA) glutamate receptors, which are responsible for mediating fast synaptic transmission in the brain, can provide insights into various cellular mechanisms. For instance, during long-term potentiation (LTP), AMPA receptors (AMPARs) containing the GluA1 subunit are trafficked to the surface of dendrites and spines (Boehm et al., 2006; Appleby et al., 2011; Diering and Huganir, 2018; Terashima et al., 2019). In contrast, AMPARs are endocytosed or internalized during long-term depression (LTD; Matsuda and Yuzaki, 2021) and upon the application of Aβ oligomers (Almeida et al., 2005; Hsieh et al., 2006; Zhang et al., 2018). AMPARs are not only trafficked between the neuronal surface and intracellular compartments by endocytosis and exocytosis, but they also move on the neuronal surface by lateral diffusion (Choquet, 2018). Surface mobility of AMPARs is thought to be important for synaptic transmission (Heine et al., 2008) and can be characterized in living neurons using single particle tracking or fluorescence recovery after photobleaching (FRAP; Ashby et al., 2006). However, to study AMPAR endocytosis, all the previously reported approaches, including immunohistochemistry and Western blotting, cannot be performed in living neurons. For example, Zhang et al. (2018) used an antibody-feeding protocol to study the effect of Aβ oligomers on GluA1 trafficking and found that surface amounts were decreased while internalized GluA1 levels increased. Similar approaches were employed to measure signaling leading to GluA2 endocytosis during cerebellar LTD (Anggono et al., 2013) and to demonstrate that oxygen deprivation specifically affected receptors containing the GluA3 subunit in hippocampal, but not cortical, neurons (Koszegi et al., 2017). Another method to measure surface AMPARs consists of a surface biotinylation assay that allows the measurement of surface and total AMPARs using Western blotting. For example, this method was used to quantify the effects of Aβ on surface amounts of GluA1-containing receptors (Zhang et al., 2018), to investigate signaling between adenosine receptors and AMPARs leading to GluA1 and GluA2 endocytosis during hypoxia (Chen et al., 2014), and to compare agonist and insulin-induced GluA1 endocytosis (Lin et al., 2000). While these approaches are very useful and have led to numerous discoveries, they require cell fixation or cell lysis and cannot be used to monitor dynamic processes in living neurons. To overcome these limitations, a new method was developed in the early 2000's, which consists of tagging AMPARs on the (extracellular) N-terminus with superecliptic pHluorin (SEP), a pH-sensitive GFP (Miesenböck et al., 1998), and permitted measurements of AMPAR exocytosis during chemical LTP (Kopec et al., 2006). This approach was originally developed to study presynaptic vesicle fusion, as the SEP fluorescence signal is completely quenched in synaptic vesicles' acidic pH, which quickly and drastically increases during vesicle fusion and neurotransmitter release (Miesenböck et al., 1998; Sankaranarayanan et al., 2000). The same principle applies to AMPARs tagged at their N-terminus, where the SEP tag on surface receptors is exposed to extracellular pH, leading to high fluorescence intensity at physiological pH and very low fluorescence intensity when receptors are inside secretory or endocytic vesicles, which contain a lower pH. This method enabled several seminal studies investigating AMPAR trafficking during synaptic plasticity (Kopec et al., 2006; Kessels et al., 2009; Fujii et al., 2018) and amyloid-beta-induced depression (Hsieh et al., 2006). However, the measurement of SEP fluorescence intensity alone cannot distinguish surface receptors from internalized receptors. Furthermore, the high variability of fluorescence intensity due to different levels of protein expression in neurons requires the use of a second fluorescent protein for normalization (Hsieh et al., 2006; Kopec et al., 2006). To make the SEP approach more broadly applicable, we developed a real-time assay of AMPAR trafficking based on fluorescence lifetime imaging (FLIM; Yasuda, 2006). FLIM measures the fluorescence lifetime, or the time delay between the excitation of a fluorescent molecule and the emission of a photon (Lakowicz, 2006). Fluorescence lifetime is a physical property, so each fluorescent molecule has its own fluorescence lifetime, which is sensitive to its physiological and chemical environment (Lakowicz, 2006). In the case of SEP, since its fluorescence intensity is sensitive to pH, we first confirmed that its fluorescence lifetime was also sensitive to pH. We found that SEP fluorescence lifetime is decreased at lower pH and can quantitatively detect very small changes in extracellular pH. Therefore, FLIM can precisely monitor AMPAR trafficking in spines and dendrites, as it can simultaneously measure surface and internalized receptors in living neurons without the use of any other normalization or special analysis.

## Results

### The fluorescence lifetime of SEP-GluA2 is sensitive to extracellular pH and insulin-induced endocytosis

**Figure 1:**
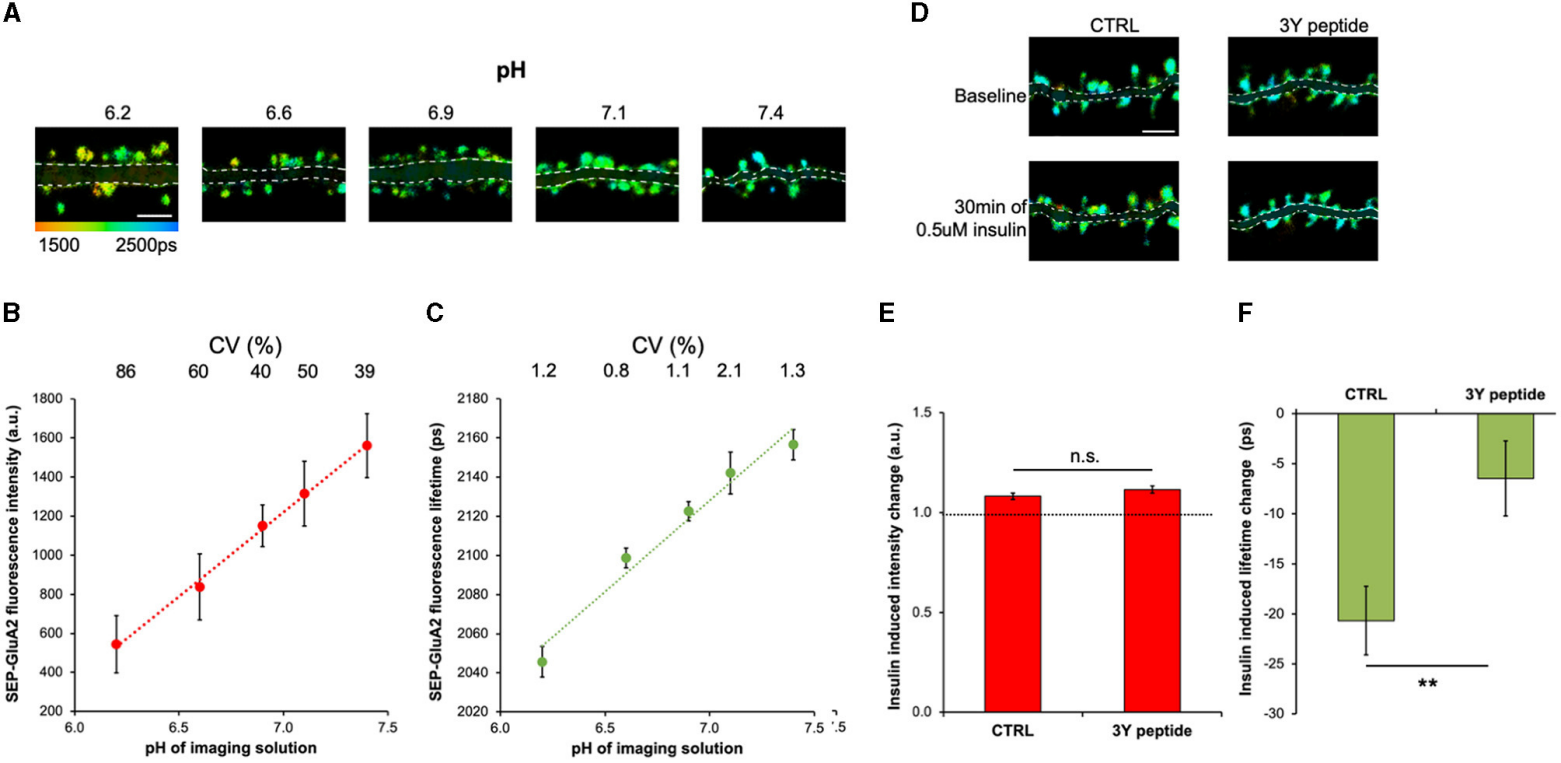
Fluorescence lifetime of SEP-GluA2 is sensitive to extracellular pH and insulin-induced endocytosis. **(A)** Representative fluorescence lifetime images of SEP-GluA2 expressing neurons in imaging solutions of different pH. The color scale indicates the fluorescence lifetime of SEP at each pixel, scale bar = 5 um. The dendrite area is shaded for clarity. **(B)** Graph of SEP-GluA2 fluorescence intensity in dendritic spines. CV is indicated above for each data point. *N* = 9–19 neurons per condition (345–791 spines per condition, data averaged by neurons). Error bars are SEM in all figures. **(C)** Graph of SEP-GluA2 fluorescence lifetime in the same spines as in **(B)**. **(D)** Fluorescence lifetime images of SEP-GluA2 before and after 0.5 μM insulin treatment for 30 min. When indicated, neurons were preincubated with 2 μM Tat-GluA2-3Y peptide (3Y) for 1 h prior to imaging, 3Y peptide was also present during imaging. **(E)** Change in SEP-GluA2 fluorescence intensity in dendritic spines for indicated conditions; *N* > 700 spines (data obtained from 16 to 25 neurons per condition). **(F)** Change in SEP-GluA2 fluorescence lifetime for indicated conditions in the same spines as in **(E)**. **p < 0.01 (unpaired *t*-test).

The fluorescence intensity of SEP is sensitive to pH (Miesenböck et al., 1998; Sankaranarayanan et al., 2000; Kopec et al., 2006); therefore, we first tested if SEP fluorescence lifetime also changes in response to different extracellular pHs. The GluA2 subunit of the AMPAR was tagged on the N-terminus with SEP, which resulted in the localization of the SEP fluorescent protein on the extracellular side of neurons and direct exposure to the imaging solution. Neurons expressing SEP-GluA2 were imaged in solutions with pH ranging from 6.2 to 7.5, and both fluorescence lifetime and intensity were measured in dendritic spines (Figure 1A). As expected, both the fluorescence intensity and lifetime were low at acidic pH, which results from fluorescence quenching (Lakowicz, 2006) and increased proportionally with increasing pH (Figures 1B, C). However, because of different expression levels of SEP-GluA2 between neurons, the coefficient of variation (CV) was 25–80 times higher in intensity-based measurements compared to lifetime measurements (Figures 1B, C). Next, we tested if SEP-GluA2 fluorescence lifetime is sensitive to AMPAR endocytosis by measuring fluorescence lifetime and intensity before and after insulin application, a manipulation shown to produce AMPAR internalization by inducing phosphorylation of three tyrosine sites in the C-terminal domain of GluA2 (Man et al., 2000; Ahmadian et al., 2004). We found that 0.5 uM insulin for 30 min reduced SEP-GluA2 fluorescence lifetime in dendritic spines (Figures 1D–F), consistent with the published effect of insulin on AMPAR endocytosis (Man et al., 2000; Ahmadian et al., 2004). The 3Y peptide, which includes the three unique GluA2 tyrosine residues, can serve as a substrate for Src kinase and effectively occlude GluA2 phosphorylation and endocytosis (Ahmadian et al., 2004). Accordingly, we did not observe any effect of insulin application in neurons incubated with the 3Y peptide (Figures 1D–F). Fluorescence intensity did not significantly change in the same spines (Figure 1E), demonstrating the power of fluorescence lifetime measurements to detect small changes.

### Elevated Aβ leads to similar reductions in SEP-GluA2 spine fluorescence lifetime in hippocampal cultures and organotypic slices

**Figure 2:**
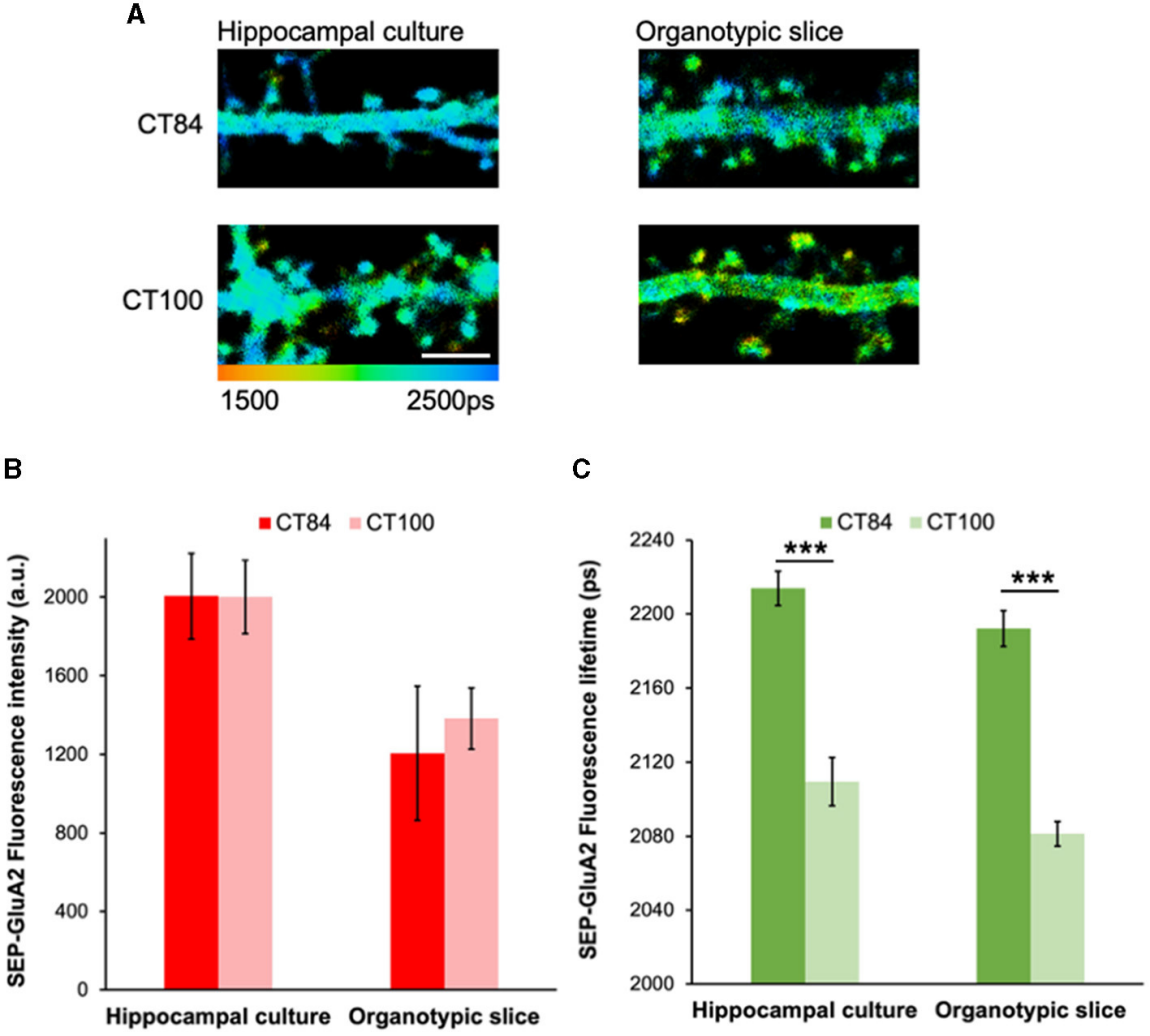
Elevated Aβ leads to similar reductions in SEP-GluA2 spine fluorescence lifetime in hippocampal cultures and organotypic slices. **(A)** Representative fluorescence lifetime images of dendrites from hippocampal cultures **(left)** or organotypic slices **(right)** expressing APP_CT84_ or APP_CT100_. The color scale indicates the fluorescence lifetime of SEP at each pixel, scale bar = 5 um. **(B)** Graph of SEP-GluA2 fluorescence intensity in dendritic spines from hippocampal cultures or organotypic slices. *N* = 14–18 neurons (389–465 spines per condition, data averaged by neurons). **(C)** Graph of SEP-GluA2 fluorescence lifetime in dendritic spines from the same neurons. ***p < 0.0001 (two-way ANOVA followed by Tukey's multiple comparison test).

Previous experiments showed that the expression of the amyloid precursor protein (APP) significantly reduced SEP-GluA2 intensity in spines and dendrites in hippocampal neurons (Hsieh et al., 2006). However, co-expression of a cytoplasmic red fluorescent protein was needed to normalize the SEP fluorescence signal and a large number of dendritic spines had to be measured (~1,000 individual data points; Hsieh et al., 2006). To test if our FLIM approach can detect Aβ-induced GluA2 endocytosis more effectively and reliably, we expressed SEP-GluA2 in two different preparations: hippocampal cultures and organotypic slices. We then measured the effect of expressing APP_CT100_, the C-terminal fragment of APP that leads to elevated levels of Aβ, and compared it to a control virus, APP_CT84_, a fragment of APP that does not change Aβ levels and was shown to have no effects on synaptic transmission (Reinders et al., 2016; Uyaniker et al., 2019; Figure 2A). We observed similar SEP fluorescence lifetimes in these two different preparations and a clear effect of APP_CT100_ expression, consistent with GluA2 endocytosis in dendritic spines (Figure 2C). In contrast, we did not observe any reductions in fluorescence intensity in these same neurons (Figure 2B). This indicates that GluA2 is similarly trafficked to dendritic spines in hippocampal cultures and organotypic slices and that elevated Aβ equally induces the endocytosis of GluA2-containing receptors in these two different neuronal preparations. Moreover, this experiment demonstrates that FLIM leads to reproducible and consistent data without needing any normalization or adjustments.

### Elevated Aβ causes different effects in GluA1- and GluA3-containing AMPARs

**Figure 3:**
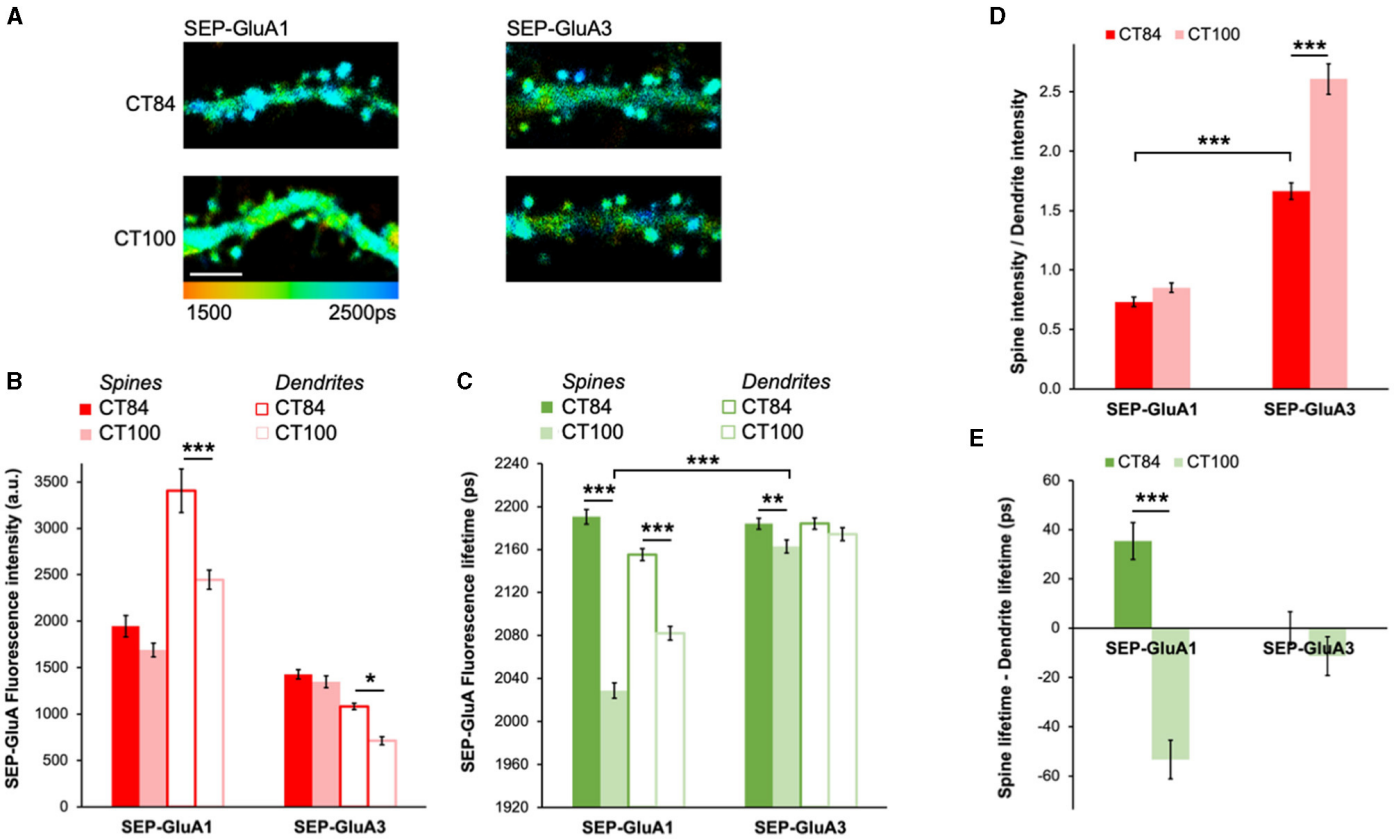
Elevated Aβ causes different effects in GluA1- and GluA3-containing AMPARs. **(A)** Representative fluorescence lifetime images of SEP-GluA1 **(left)** or SEP-GluA3 **(right)** expressing APP_CT84_ or APP_CT100_. The color scale indicates the fluorescence lifetime of SEP at each pixel, scale bar = 5 μm. **(B)** Graph of SEP-GluA1 and SEP-GluA3 fluorescence intensity in spines and dendrites of neurons expressing APP_CT84_ or APP_CT100_. *N* > 210 spines and adjacent dendritic regions of interest (ROIs). Data are obtained from 7 to 12 neurons per condition. *p < 0.05, ***p < 0.0001 (two-way ANOVA followed by Tukey's multiple comparison test). **(C)** Graph of SEP-GluA1 and SEP-GluA3 fluorescence lifetime in the same ROIs. **p < 0.01, ***p < 0.0001 (two-way ANOVA followed by Tukey's multiple comparison test). **(D)** Graph of spine/dendrite fluorescence intensity ratio in neurons expressing SEP-GluA1 and SEP-GluA3, using data shown in **(B)**, calculated for each individual spine-dendrite pair. ***p < 0.001 (2-way ANOVA followed by Tukey's multiple comparison test). **(E)** Graph of fluorescence lifetime difference between spines and dendrites in the same neurons using data shown in **(C)**, calculated for each individual spine-dendrite pair. ***p < 0.001 (two-way ANOVA followed by Tukey's multiple comparison test).

AMPARs are composed of four subunits and are mostly found as pairs of GluA1 and GluA2 (GluA1/2) or GluA2 and GluA3 (GluA2/3) dimers in pyramidal neurons (Wenthold et al., 1996). To test if GluA1/2 or GluA2/3 receptors are differentially affected by elevated levels of Aβ, we expressed SEP-GluA1 and SEP-GluA3 along with either APP_CT100_ or the APP_CT84_ control (Figure 3A). Similarly, as with SEP-GluA2 (Figure 2), APP_CT100_ did not lead to any significant reductions in fluorescence intensity in spines of neurons expressing SEP-GluA1 or SEP-GluA3 as compared with neurons expressing APP_CT84_ (Figure 3B). Interestingly, in dendrites, a significant decrease in fluorescence intensity was seen in APP_CT100_-expressing neurons for both SEP-GluA1 and SEP-GluA3 (Figure 3B). For GluA1-containing receptors, the effect of elevated Aβ on SEP-GluA1 fluorescence lifetime was very obvious both in spines and dendrites (Figure 3C). However, in SEP-GluA3-expressing neurons, compared to APP_CT84_ control neurons, APP_CT100_ expression induced only a small decrease in fluorescence lifetime in spines and no significant effect in dendrites (Figure 3C).

To gain more insight into AMPAR trafficking, we looked at the spine intensity to dendrite intensity ratio, which can be used as a proxy for receptor enrichment in spines (Kopec et al., 2006). Elevated Aβ significantly increased the spine/dendrite ratio in GluA3-containing receptors (Figure 3D), indicating that SEP-GluA3 fluorescence intensity in dendrites was significantly more reduced than in spines. In contrast, elevated Aβ did not affect GluA1 spine enrichment (Figure 3D). Comparing the spine/dendrite ratio for GluA1 and GluA3 in control conditions (APP_CT84_), we found that GluA3-containing receptors were significantly more enriched in spines than GluA1-containing receptors, consistent with previous literature (Kopec et al., 2006). We also looked at the difference between spine and dendrite lifetime, which gives an indication of the relative proportion of surface receptors in spines vs. dendrites. For SEP-GluA1, this difference was positive in control conditions, suggesting that there are more GluA1-containing receptors at the surface in spines than in dendrites (Figure 3E). APP_CT100_ caused a significant reduction in the difference in lifetime for SEP-GluA1, indicating an increase in internalized receptors in spines specifically (Figure 3E). For SEP-GluA3, the difference in lifetime was close to 0 and was not affected by APP_CT100_ expression, suggesting that there is a similar surface/internalized ratio in spines and dendrites and that this ratio is not affected by elevated Aβ (Figure 3E).

### AMPAR trafficking in hippocampal cultures from APP/PS1 mice

**Figure 4:**
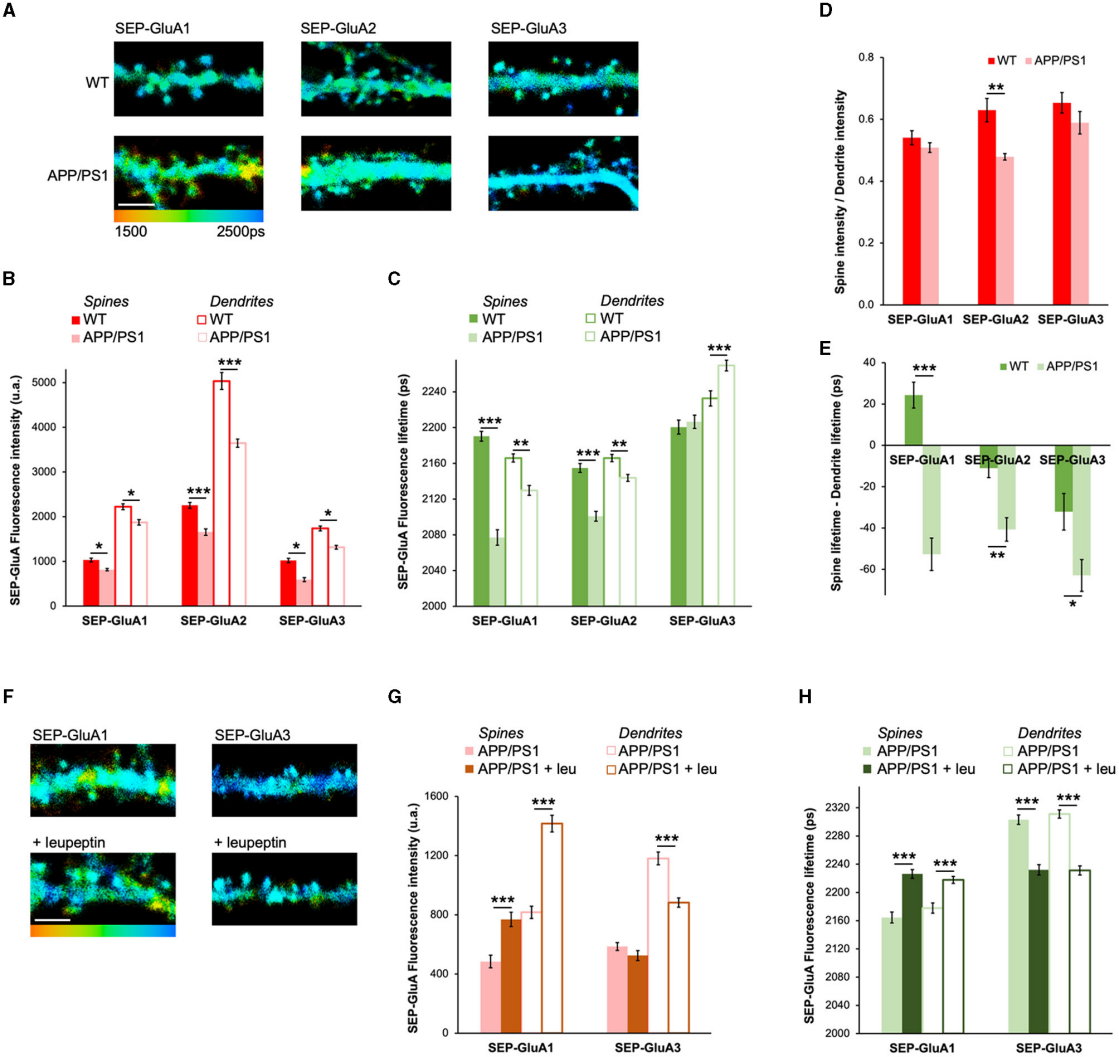
AMPAR trafficking in hippocampal cultures from APP/PS1 mice. **(A)** Representative fluorescence lifetime images of SEP-GluA1 **(left)**, SEP-GluA2 **(middle)**, or SEP-GluA3 **(right)** from WT or APP/PS1 neurons from hippocampal cultures. The color scale indicates the fluorescence lifetime of SEP at each pixel, scale bar = 5 um. **(B)** Graph of fluorescence intensity in WT or APP/PS1 neurons expressing SEP-GluA1, SEP-GluA2, or SEP-GluA3; spines and adjacent dendrite ROIs. *N* > 388 spines and adjacent dendritic ROIs. Data are obtained from 10 to 15 neurons per condition. ***p < 0.0001, *p < 0.05 (two-way ANOVA followed by Tukey's multiple comparison test). **(C)** Graph of fluorescence lifetime for SEP-GluA1, SEP-GluA2, or SEP-GluA3, in the same neurons as in **(B)**. ***p < 0.0001, **p < 0.01 (two-way ANOVA followed by Tukey's multiple comparison test). **(D)** Graph of spine/dendrite fluorescence intensity ratio in neurons expressing SEP-GluA1, SEP-GluA2, or SEP-GluA3, using data shown in **(B)**, calculated for each individual spine-dendrite pair. **p < 0.01 (two-way ANOVA followed by Tukey's multiple comparison test). **(E)** Graph of fluorescence lifetime difference between spines and dendrites in the same neurons using data shown in **(C)**, calculated for each individual spine-dendrite pair. *p < 0.05, **p < 0.01. ***p < 0.0001 (two-way ANOVA followed by Tukey's multiple comparison test). **(F)** Representative fluorescence lifetime images of SEP-GluA1 without (top left) and with **(bottom left)** leupeptin or of SEP-GluA3 without (top right) and with (bottom right) leupeptin. Color scale indicates the fluorescence lifetime of SEP at each pixel, scale bar = 5 um. **(G)** Graph of fluorescence intensity of SEP-GluA1 or SEP-GluA3-expressing neurons in spines and dendrites of APP/PS1 mice with or without leupeptin. *N* > 210 spines and adjacent dendritic ROIs. Data are obtained from 9 to 19 neurons per condition. ***p < 0.0001 (two-way ANOVA followed by Tukey's multiple comparison test). **(H)** Graph of the fluorescence lifetime of SEP-GluA1 or SEP-GluA3 in the same neurons. ***p < 0.0001 (two-way ANOVA followed by Tukey's multiple comparison test).

To study the effect of Aβ on AMPAR trafficking in more detail, we used hippocampal cultures made from APP/PS1 mice and their WT littermates. APP/PS1 mice are double transgenic Alzheimer's disease (AD) model mice expressing human APP and presenilin 1 with familial mutations causing early disease onset (Jankowsky et al., 2004). In these AD model mice, spine density was shown to be significantly reduced in both cortical and hippocampal primary cultures, indicating early synaptic deficits (Priller et al., 2009; Kashyap et al., 2019). Since AMPAR removal most likely mediates synaptic loss in amyloidosis AD models (Hsieh et al., 2006), we assessed AMPAR trafficking in APP/PS1 neurons. To do so, we expressed SEP-GluA1, SEP-GluA2, or SEP-GluA3 in hippocampal cultures from either WT or APP/PS1 mice (Figure 4A). We found that the fluorescence intensity in spines and dendrites of neurons expressing SEP-GluA1, SEP-GluA2, and SEP-GluA3 was significantly reduced in APP/PS1 neurons compared to WT controls, indicating significantly lower amounts of all AMPARs (Figure 4B). Additionally, we found that the fluorescence lifetime in spines and dendrites for APP/PS1 neurons expressing SEP-GluA1 and SEP-GluA2 was significantly decreased (Figure 4C). This is consistent with relatively more GluA1/2 receptors in endocytic vesicles in APP/PS1 neurons compared to WT neurons. Surprisingly, for SEP-GluA3, we saw a significant fluorescence lifetime increase in dendrites and no change in spines (Figure 4C). This fluorescence lifetime increase suggests that there are more surface receptors and fewer internalized GluA3-containing receptors in APP/PS1 dendrites compared to WT neuron dendrites.

Using the spine/dendrite ratio to examine AMPAR enrichment in spines, we found that neurons expressing SEP-GluA2 had a significant decrease in this ratio for APP/PS1 compared to WT, indicating that the enrichment of GluA2-containing receptors was reduced in APP/PS1 neurons, possibly due to more endocytosis in spines (Figure 4D). We also looked at the difference between spine and dendrite lifetime, which is similar to what we found by acutely increasing Aβ with APP_CT100_ in Figure 3. For SEP-GluA1, this difference was positive in WT neurons and changed to negative in APP/PS1 neurons. This suggests that there are more surface GluA1-containing receptors in the spines of WT mice compared to dendrites and more surface receptors in dendrites of APP/PS1 neurons (Figure 4E). For neurons expressing SEP-GluA2 or SEP-GluA3, this difference between spine and dendrite lifetime also decreased, suggesting reductions in surface receptors at spines, specifically in APP/PS1 neurons (Figure 4E).

For GluA1- and GluA2-containing AMPARs, we see consistent results using either APP_CT100_ to express Aβ (Figures 2, 3) or in APP/PS1 neurons (Figure 4). However, the trafficking of GluA3-containing receptors is not clear and, importantly, seems inconsistent with a previous study indicating the requirement of the GluA3 subunit for Aβ-induced depression and memory deficits in APP/PS1 mice (Reinders et al., 2016). A possible explanation for this discrepancy is that GluA2/3 AMPARs are more susceptible to lysosomal degradation than GluA1/2 AMPARs (Kessels et al., 2009). Therefore, SEP-GluA3 subunits may be degraded too quickly for us to measure their trafficking to intracellular vesicles. In order to test this idea, we used leupeptin, a lysosomal protease inhibitor that prevents degradation of internalized AMPAR receptors up to 60 min after their endocytosis (Ehlers, 2000). APP/PS1 neurons expressing SEP-GluA1 or SEP-GluA3 were treated with leupeptin and compared with control neurons (Figure 4F). In APP/PS1 neurons expressing SEP-GluA1, leupeptin induced a significant increase in both fluorescence intensity and lifetime compared to untreated neurons (Figures 4G, H), which is consistent with increased amounts of surface GluA1-containing receptors both in spines and dendrites. Strikingly, we observed the opposite effect in neurons expressing SEP-GluA3. In APP/PS1 cultures treated with leupeptin, SEP-GluA3 fluorescence intensity in dendrites was decreased, as was SEP-GluA3 fluorescence lifetime in both spines and dendrites (Figures 4G, H). Therefore, by blocking lysosomal degradation, we were able to see results consistent with endocytosis of GluA3-containing AMPARs in APP/PS1 mouse neurons. Moreover, the fact that the same treatment instead increased surface GluA1-containing AMPARs suggests largely different degradation dynamics of GluA1- and GluA3-containing AMPARs, highlighting the opposite trafficking properties of these two types of AMPARs.

### PP1 expression mimics Aβ-induced synaptic depression by driving GluA1 endocytosis

**Figure 5:**
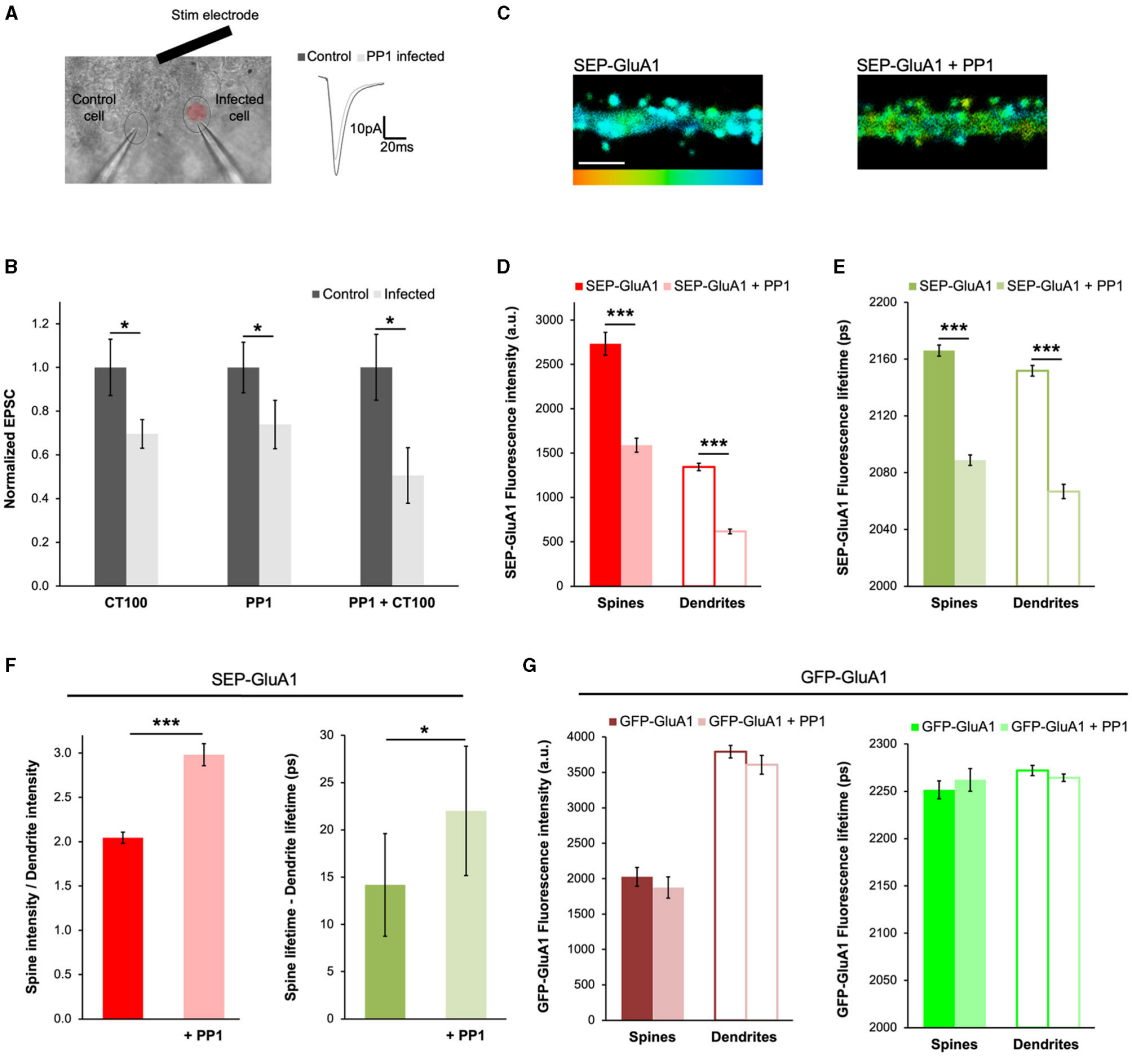
PP1 expression mimics Aβ-induced synaptic depression by driving GluA1 endocytosis. **(A)** Experimental setup of electrophysiology experiments showing a DIC picture of a slice and a pair of patch-clamped neurons (a control cell and one infected with PP1; left), and example traces (mean of 50 consecutive trials) of such a recording **(right)**. **(B)** Graph showing the normalized EPSC from cells infected with APP_CT84_, APP_CT100_, or PP1. Responses normalized to uninfected controls (dark gray). *N* = 11–17 paired recordings per condition (obtained from 6 to 13 slices), *p < 0.05 (paired *t*-test). **(C)** Representative fluorescence lifetime images of SEP-GluA1 expressed alone **(left)** or co-expressed with PP1-mCherry **(right)**. The color scale indicates the fluorescence lifetime of SEP at each pixel, scale bar = 5 um. **(D)** Graph of the fluorescence intensity of SEP-GluA1 or SEP-GluA1 + PP1-mCherry expressing neurons. *N* > 408 spines and adjacent dendritic ROIs. Data are obtained from 7 to 10 neurons per condition. ***p < 0.0001 (two-way ANOVA followed by Tukey's multiple comparison test). **(E)** Graph of the fluorescence lifetime of SEP-GluA1 or SEP-GluA1 + PP1-mCherry in the same neurons. ***p < 0.0001 (two-way ANOVA followed by Tukey's multiple comparison test). **(F)** Graph of spine/dendrite fluorescence intensity ratio **(left)** or the fluorescence lifetime difference between spines and dendrites **(right)** in neurons expressing SEP-GluA1 or SEP-GluA1 + PP1-mCherry, calculated for each individual spine-dendrite pair. *p < 0.05, ***p < 0.0001 (Kolmogorov–Smirnov test). **(G)** Graph of fluorescence intensity **(left)** or lifetime **(right)** from neurons expressing GFP-GluA1 or GFP-GluA1 + PP1-mCherry. *N* > 140 spines and adjacent dendritic ROIs. Data are obtained from 4 to 7 neurons per condition.

To further demonstrate the applicability of our approach to different contexts, we decided to test if the expression of PP1 had any effects on the trafficking of GluA1-containing AMPARs. PP1 is a protein phosphatase that participates in LTD (Mulkey et al., 1993; Morishita et al., 2001; Aow et al., 2015) and was recently found to be activated by APP_CT100_ expression (Dore et al., 2021). During LTD, PP1 dephosphorylates GluA1 subunits at Ser845 (Lee et al., 2000), which is thought to induce its endocytosis (Guntupalli et al., 2017). To study how Aβ or PP1 affects synaptic transmission, we sparsely infected organotypic hippocampal slices with Sindbis viruses expressing APP_CT100_ or the catalytic subunit of PP1 (PP1_cat_, designated as PP1) and 18–24 h later obtained whole-cell recordings simultaneously from pairs of infected and uninfected neurons (Figures 5A, B, as in Dore et al., 2021). As expected (Kessels et al., 2013; Dore et al., 2021), neurons expressing APP_CT100_ displayed depressed synaptic transmission compared to uninfected control neurons (Figure 5B). Interestingly, the expression of PP1 by itself or along with Aβ produced a similar effect (Figure 5B), suggesting that these processes may be mediated by common signaling pathways. Additionally, we found that PP1 expression led to a significant reduction in both SEP-GluA1 fluorescence intensity and lifetime, consistent with SEP-GluA1 endocytosis (Figures 5C–E). The ratio of the spine to dendrite intensity increased with PP1 expression, indicating that fluorescence intensity in dendrites was significantly more affected (Figure 5F). Accordingly, we also saw an increase in the difference between the spine and dendrite lifetime (Figure 5F), suggesting that more AMPARs are being removed from the dendrites compared to the spines. In these experiments, the PP1 protein expressed was tagged with mCherry, which could induce FRET between GluA1 and PP1 if these proteins were close enough. However, the SEP tag on GluA1 is extracellular and PP1 is a cytoplasmic protein, so this possibility is very unlikely. Nevertheless, to make sure that the change in SEP fluorescence lifetime is not due to proximity with mCherry, we expressed GFP-tagged GluA1 (at its N-terminus, exactly like the SEP tag) and the same PP1-mCherry (Figure 5G). We found that PP1-mCherry had no effect on GFP-GluA1 fluorescence intensity or lifetime, confirming that these proteins are not close enough to allow FRET between GFP and mCherry. This indicates that the changes we measured using SEP-GluA1 (Figures 5C–E) are due to the endocytosis of GluA1-containing AMPARs.

## Discussion

Changes in transmembrane receptor trafficking are occurring constantly and are essential for numerous signaling mechanisms, including synaptic plasticity, hypoxia, and several disease conditions. To investigate these changes, methods that can monitor receptor trafficking from intracellular vesicles to the cell surface (and vice versa) in living neurons are needed. Because FLIM can measure both surface and internalized receptors simultaneously, we were able to measure AMPAR endocytosis in several different contexts, including insulin application, elevated Aβ (by using the APP_CT100_ virus and in APP/PS1 mouse cultures), and PP1 expression. Moreover, we demonstrated that these experiments did not require any normalization (as the fluorescence lifetime is not affected by intensity) and led to reliable results in both primary hippocampal neurons and organotypic slices. Importantly, we used this approach to reveal differences in how three AMPAR subunits (GluA1, GluA2, and GluA3) are affected by viral expression of APP_CT100_ and in APP/PS1 mouse neurons.

Using the *in vitro* AD model of acutely expressing APP_CT100_, we found that SEP-GluA1 and SEP-GluA3 fluorescence intensity were significantly reduced in dendrites but not in spines. In contrast, in cultures from APP/PS1 mice, fluorescence intensity decreased in both the spines and dendrites of neurons expressing SEP-GluA1, SEP-GluA2, and SEP-GluA3, indicating that chronic Aβ exposure leads to robust reductions in all AMPARs. Looking at fluorescence lifetime, we found that APP_CT100_ expression leads to considerable reductions in SEP-GluA1 and SEP-GluA2 fluorescence lifetime in dendritic spines and dendrites. Similar results were obtained when comparing APP/PS1 and WT neurons. For SEP-GluA3, APP_CT100_ induced a small but significant fluorescence lifetime decrease in dendritic spines and no change in dendrites. In APP/PS1 neurons, we did not see any decrease in fluorescence lifetime for SEP-GluA3 but instead an increase in dendrites. This suggests that Aβ would have minimal effects on GluA3-containing AMPARs. As GluA3 was reported to be required for Aβ's effects on synaptic transmission, spine density, and memory in APP/PS1 mice (Reinders et al., 2016), this result is unexpected. However, the fact that SEP-GluA3 fluorescence intensity decreased in APP/PS1 cultures suggests that these receptors were removed, and the increase in fluorescence lifetime in dendrites suggests that there is an even larger proportion of receptors at the cell surface. This would be consistent with Aβ leading to complete degradation of GluA3-containing AMPARs in intracellular vesicles and with the previous literature indicating that GluA2/3 subunits are ~6 times more likely to be targeted for lysosomal degradation than GluA1 subunits (Kessels et al., 2009). Our results in APP/PS1 neurons treated with leupeptin expressing SEP-GluA1 suggest that these receptors are degraded slowly and that blocking degradation increases their amount at the surface. In contrast, for GluA3-containing receptors, leupeptin resulted in more internalized receptors, which could be because this treatment permitted visualization of these receptors before their accelerated degradation.

APP_CT100_ expression also leads to the depression of synaptic transmission (Kamenetz et al., 2003; Kessels et al., 2013; Dore et al., 2021) (Figure 5), which is mediated by AMPAR removal. Interestingly, we found that the expression of the phosphatase PP1, which is required for LTD (Mulkey et al., 1993), also depressed synaptic transmission. As PP1 is known to dephosphorylate GluA1, the trafficking of this subunit was assessed using our FLIM approach. We saw that PP1 expression drastically decreased surface amounts of GluA1 in spines and even more so in dendrites. As electrically evoked responses are generated only by synaptic receptors, our FLIM experiments provide more information on PP1 actions on GluA1-containing AMPARs.

Altogether, this study suggests that measuring the trafficking of SEP-tagged transmembrane receptors using FLIM is a reliable approach that does not need any signal normalization and, most importantly, can help uncover precise signaling mechanisms.

## Materials and methods

### Animals

C57BL/6J mice were used for most experiments (#000664-JAX) and referred to as wild-type (WT) mice. For Figure 4, APP/PS1 Alzheimer's model mice (Jankowsky et al., 2004; #034832-JAX) and their WT littermates were used. The mice were kept in the UCSD School of Medicine facility on a 12-h light–dark cycle, given *ad libitum* access to food and water, and their genotypes were confirmed using genotyping PCR. All animal procedures were approved by UCSD's IACUC.

### Primary hippocampal neuronal cultures

Primary hippocampal neuronal cultures were made using P0–P1 mice pups according to previously published protocols (Dore et al., 2021). Briefly, hippocampi were dissected using ice-cold dissection media, cut into small pieces using a scalpel, resuspended in dissociation media [dissection media with 2 mM L-cysteine hydrochloride (Sigma), 10 mg/mL of papain (Sigma), pH 7.4], and incubated for 12 min at 37°C. After cell filtration and precipitation, the neurons were resuspended in plating media [Neurobasal-A (Gibco), 10% FBS, 0.5% Pen/Strep (Gibco), and 0.25% GlutaMAX (Gibco)] at a concentration of 1–2 × 10^6^ cells/mL and plated on 18 mm PDL-coated glass coverslips (Neuvitro). At 7–10 DIV, the neurons were transfected using ~2 μg total DNA and ~4 μL of lipofectamine-2000 (Thermo Fisher) per well. SEP-GluA1 and SEP-GluA2 were gifts from Roberto Malinow (Addgene plasmids # 24000 and # 24002; RRID:Addgene_24000, RRID:Addgene_24002, respectively). SEP-GluA3 was a gift from Helmut Kessels (Renner et al., 2017). For Figures 2, 3, 18–24 h before imaging, the neurons were infected with 1–2 μL of Sindbis virus to express APP_CT84_ or APP_CT100_ as in Dore et al. (2021). For Figure 5, the PPP1CA sequence from the Addgene plasmid # 155843 (RRID:Addgene_155843, a gift from Eugene Yeo) was tagged with mCherry at its N-terminus and cloned into a PCI vector (for FLIM experiments) or a Sindbis vector (for electrophysiology) using Gibson assembly. GFP-GluA1 was also a gift from Roberto Malinow (Kopec et al., 2006). To account for biological variables, all experiments were conducted in at least three different neuronal culture preparations.

### Organotypic slices

Organotypic hippocampal slices were prepared from P5 to P7 C57BL/6J mouse pups as described (Stoppini et al., 1991) and maintained for 8–12 days before infecting them with Sindbis viruses. The slices were infected in the CA1 region using an injection pipette and a Picospritzer 18–24 h before imaging (Figure 2) or electrophysiological recordings (Figure 5). For Figure 2, Sindbis viruses expressing either APP_CT84_ or APP_CT100_ were mixed with a SEP-GluA2 Sindbis virus (pSIN REP5-GFP-GluR2 (Q), a gift from Roberto Malinow (Addgene plasmid # 24003; RRID:Addgene_24003). For Figures 5A, B, the slices were infected with Sindbis viruses expressing either: APP_CT100_, PP1-mCherry, or APP_CT100_ and PP1 by means of a double promoter. Similarly, as with the experiments conducted in primary neurons, experiments in organotypic slices were replicated in three independent slice culture preparations.

### FLIM

FLIM was performed using a SliceScope Two-Photon Microscope (Scientifica, UK) with excitation from a Chameleon Ultra II IR laser tuned to 900 nm. Fluorescence emission was captured using a hybrid PMT detector (HPM-100-10, Becker and Hickl) and a GFP emission filter (ET 515/50, Chroma). The arrival time of each photon is calculated with a TCSPC (time-correlated single photon counting) module (SPM-150, Becker and Hickl). The FLIM acquisition software uses this information to construct a fluorescence decay trace, which indicates the number of photons detected in each time bin and is used to calculate the fluorescence lifetime (see Analysis section below). To minimize phototoxicity and photobleaching, a maximum of 3 mW of laser power was used for excitation, scanning speed was high (pixel dwell time of 3.2 μs) and image acquisition time was 120 s maximum per image. See Dore et al. (2021) for more information on FLIM acquisition and analysis parameters. Primary hippocampal neurons were imaged at 14–21 DIV in a circulating perfusion of an HBSS-based imaging solution comprised of: 0.87x HBSS, 10 mM HEPES, 2 mM Glucose, 1 mM MgCl_2_, and 1.2 mM CaCl_2_. For all experiments in this study, living neurons were used. For Figures 1A–C, HEPES was replaced with MES [membrane impermeable acid, (Sigma)] to obtain solutions with pH levels varying from 6.2 to 7.4. For experiments shown in Figures 1D–F, after imaging neurons in regular HBSS solution, perfusion was switched to the same solution supplemented with 0.5 μM insulin (Sigma). When indicated, the neurons were preincubated with the Tat-GluA2-3Y peptide (AnaSpec) for 1 h prior to imaging at a final concentration of 2 μM, and this peptide was also added to the imaging solution. Similarly, for experiments shown in Figures 4F–H, 20 μM leupeptin (AG Scientific) was preincubated for 3 h and also present during imaging.

### Electrophysiology

Whole-cell recordings were performed in hippocampal organotypic slices infected with Sindbis viruses, as described above in the Organotypic Slices section. The slices were transferred to a recording chamber with a 1.5–2.0 ml/min flow of oxygenated artificial cerebrospinal fluid (aCSF), containing 119 mM NaCl, 2.5 mM KCl, 26 mM NaHCO_3_, 1 mM NaH_2_PO_4_, 10 mM glucose, 4 mM CaCl_2_, 4 mM MgCl_2_, 10 μM gabazine, and 4 μM 2-chloroadenosine (pH 7.4). Healthy control and infected pyramidal neurons in CA1 were found using differential interference contrast and fluorescence microscopy. Pipettes with 3–5 MΩ resistance were filled with an internal solution containing (in mM): 115 cesium methanesulfonate, 20 CsCl, 10 HEPES, 2.5 MgCl_2_, 4 Na_2_ATP, 0.4 Na_3_GTP, 10 sodium phosphocreatine, and 0.6 EGTA at a pH of 7.3 and utilized to obtain whole-cell recordings with an Axopatch-1D amplifier (Molecular Devices). A stimulating electrode [contact Pt/Ir cluster bipolar electrode (Frederick Haer)] was placed in Stratum Radiatum ~200–300 μm down the apical dendrite to evoke AMPAR-mediated excitatory post-synaptic currents (EPSCs) under voltage-clamp at a holding potential of −60 mV. Evoked responses of each recorded cell were analyzed using Igor Pro software, and amplitudes were averaged from 30 to 100 sweeps.

### Data analysis and statistics

Fluorescence lifetime images were generated with SPCImage (Becker and Hickl), which calculates the fluorescence lifetime from the acquired fluorescence decay traces at each pixel. For all experiments, a binning factor between 2 and 6 pixels, a minimum threshold of 10 photons at the peak time bin, a single exponential model, and the same calculated instrumental response function were used. The FLIM images shown in all figures were processed in SPCImage and consisted of the fluorescence lifetime (color-coded value) merged with the intensity information (total number of photons) at each pixel. For further analysis, each FLIM image was exported as a matrix containing lifetimes, photon counts, and goodness-of-fit values (chi-square) and analyzed blindly to condition using a custom MATLAB script; refer to Dore et al. (2015) for details. All data are presented as mean ± standard error of the mean (SEM). Statistics were conducted using Prism 9 software. The unpaired *t*-test or the Kolmogorov–Smirnov test was used when comparing only two groups. In Figure 5B, a paired *t*-test was used. For all other experiments comparing multiple groups, a two-way ANOVA followed by Tukey's multiple comparison test was used to determine statistical significance.

## Data availability statement

The original contributions presented in the study are included in the article/supplementary material, further inquiries can be directed to the corresponding author.

## Ethics statement

The animal study was approved by UCSD's Institutional Animal Care and Use Committee (IACUC). The study was conducted in accordance with the local legislation and institutional requirements.

## Author contributions

KP: Data curation, Investigation, Writing – original draft. ET: Data curation, Writing – original draft. JS: Data curation, Investigation, Writing – original draft. TC: Data curation, Writing – original draft. KD: Conceptualization, Data curation, Funding acquisition, Investigation, Methodology, Project administration, Supervision, Writing – original draft, Writing – review & editing.
